# Norvaline is accumulated after a down-shift of oxygen in *Escherichia coli W3110*

**DOI:** 10.1186/1475-2859-7-30

**Published:** 2008-10-21

**Authors:** Jaakko Soini, Christina Falschlehner, Christina Liedert, Jörg Bernhardt, Jussi Vuoristo, Peter Neubauer

**Affiliations:** 1 Bioprocess Engineering Laboratory, Department of Process and Environmental Engineering and Biocenter Oulu, University of Oulu, P. O. Box 4300, FI-90014 Oulu, Finland; 2 Department of Microbial Physiology, Institute of Microbiology, Ernst-Moritz-Arndt-University Greifswald, Friedrich-Ludwig-Jahn-Str. 15, D-17489 Greifswald, Germany; 3 Biocenter Oulu, University of Oulu, P. O. Box 5000, FI-90014 Oulu, Finland; 4 Department of Bioprocess Technology, Institute of Biotechnology, Technische Universität Berlin, Ackerstr. 71-76, D-13355 Berlin, Germany

## Abstract

**Background:**

Norvaline is an unusual non-proteinogenic branched-chain amino acid which has been of interest especially during the early enzymological studies on regulatory mutants of the branched-chain amino acid pathway in *Serratia marcescens*. Only recently norvaline and other modified amino acids of the branched-chain amino acid synthesis pathway got attention again when they were found to be incorporated in minor amounts in heterologous proteins with a high leucine or methionine content. Earlier experiments have convincingly shown that norvaline and norleucine are formed from pyruvate being an alternative substrate of α-isopropylmalate synthase, however so far norvaline accumulation was not shown to occur in non-recombinant strains of *E. coli*.

**Results:**

Here we show that oxygen limitation causes norvaline accumulation in *E. coli *K-12 W3110 during grow in glucose-based mineral salt medium. Norvaline accumulates immediately after a shift to oxygen limitation at high glucose concentration. On the contrary free norvaline is not accumulated in *E. coli *W3110 in aerobic cultures. The analysis of medium components, supported by transcriptomic studies proposes a purely metabolic overflow mechanism from pyruvate into the branched chain amino acid synthesis pathway, which is further supported by the significant accumulation of pyruvate after the oxygen downshift. The results indicate overflow metabolism from pyruvate as necessary and sufficient, but deregulation of the branched chain amino acid pathway may be an additional modulating parameter.

**Conclusion:**

Norvaline synthesis has been so far mainly related to an imbalance of the synthesis of the branched chain amino acids under conditions were pyruvate level is high. Here we show that simply a downshift of oxygen is sufficient to cause norvaline accumulation at a high glucose concentration as a consequence of the accumulation of pyruvate and its direct chain elongation over α-ketobutyrate and α-ketovalerate.

Although the flux to norvaline is low, millimolar concentrations are accumulated in the cultivation broth, which is far above the level which has been discussed for being relevant for misincorporation of norvaline into recombinant proteins. Therefore we believe that our finding is relevant for recombinant protein production but also may even have implications for the physiology of *E. coli *under oxygen limitation in general.

## Background

Norvaline belongs to the group of non-usual amino acid analogs that may be formed under certain circumstances as byproducts of the branched-chain amino acid biosynthetic pathway in *E. coli *and other Gram-negative microorganisms. These amino acids can accumulate and are secreted under certain cultivation conditions.

Historically, norvaline has been reported in one example to be a natural component of an antifungal peptide produced by *Bacillus subtilis *[1]. Later it was found as a side product in isoleucine overproducing regulatory mutants of *Serratia marcescens *which initiated a number of physiological studies by Chibata and colleagues [2-7]. Recently, the formation of modified branched chain amino acids has got increasing attention as these amino acids appeared to be incorporated in certain recombinant proteins produced in *E. coli*, e.g. β-methylnorleucine into a recombinant hirudin [8-10], norleucine into recombinant human brain derived neurotrophic factor [11], interleukin 2 [12-14], and bovine somatotropin [15], and norvaline into recombinant hemoglobin [16].

Incorporation of the modified amino acids into proteins occurs over mis-aminoacylation of tRNAs; namely of leucine-tRNA by norvaline, methionine-tRNA by norleucine [11-23] and to a low probability by norvaline, and ile-tRNA by β-methylnorleucine.

The incorporation of these unusual amino acids in recombinant proteins has been assigned to conditions which derepress the branched-chain amino acid pathway, which can occur due to strong expression of leucine-rich proteins [14]. Subsequently derepression of the pathway occurs by the low concentration of the proteinogenic amino acid leucine.

**Figure 1:**
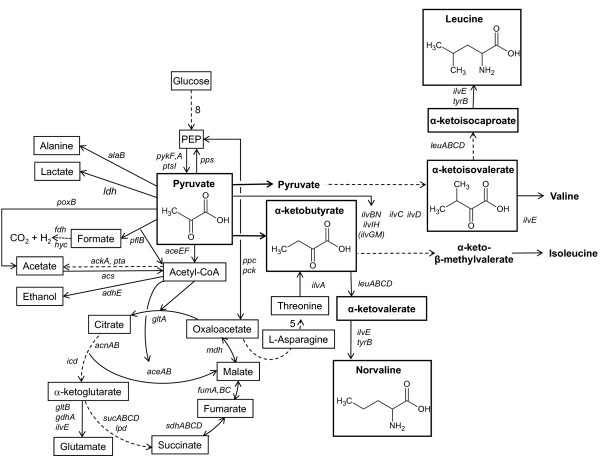
**Schematic view of the branched chain amino acid pathway with the proposed pathway for norvaline synthesis from pyruvate in relation with glycolysis and the tricarbonic acid cycle in *E. coli*.** Overflow metabolites and anaerobic routes are included. For simplification some metabolic pathways with more steps are illustrated by an interrupted line. Numbers relate to the amount of enzymatic reactions. Enzymes are indicated by their gene names.

The synthesis of the modified amino acids of the branched-chain amino acid synthesis pathway derives from the isoleucine route which starts with α-ketobutyrate and their synthesis is caused by the low specificity of the enzymes of the branched-chain amino acid synthesis pathway (including the gene products of the *ilv *and *leu *operons) for α-keto acids. A probable scheme of the synthesis of the modified amino acids has been published by Apostol et al. [16] and is shown for norvaline in Figure 1.

The branched-chain amino acid pathway for the synthesis of the three amino acids isoleucine, valine and leucine is closely interconnected to the glycolytic pathway. The major glycolysis-based metabolic intermediates involved are pyruvate and acetyl-CoA. α-ketobutyrate which is the substrate for the isoleucine pathway is synthesized from oxaloacetate over a number of intermediates, including L-aspartate, homoserine, and threonine. However, as a shorter and probable alternative for accumulation of α-ketobutyrate during conditions where the modified amino acids accumulate transiently, the direct extension of the carbon chain of pyruvate by the enzymes of the *leu *operon was proposed by Bogosian et al. [15] supported by kinetic data for α-isopropylmatate synthase from *Salmonella typhimurium *[24] and *Serratia marcescens *[2].

The specific feature of the branched amino acid pathway which has made this route interesting for a number of molecular-physiological and biochemistry studies is (i) the interactive metabolic feed-back regulation of synthetic enzymes by accumulation of one or more of the amino acids. In consequence amino acid starvation responses can be provoked by over-accumulation of one of the amino acids, which leads to suppression of the synthesis of the others. A well established example is the induction of the stringent response in *E. coli *K-12 strains by addition of valine which results in isoleucine starvation [25,26]. (ii) The expression levels of the enzymes of the branched-chain amino acid pathway are regulated in dependence on the cellular concentration of each of the branched chain amino acids by the interactive repression of mRNA synthesis of the operons of the metabolic pathway by attenuation. (iii) Finally, a specific characteristic of the enzymes of this pathway is their low substrate specificity. Most of the enzymes can accept other than the main α-ketoacids as substrate. As a result, also other modified amino acids can be synthesized. Norvaline and norleucine directly diverge from α-ketobutyrate by chain elongation through the action of α-isopropylmalate synthase (α-IPMS), α-isoprolylmalate isomerase, β-isoprolylmalate dehydrogenase and following transamination. For this reaction accumulation of α-ketobutyrate is a prerequisite, because the affinity of α-IPMS for α-ketobutyrate is an order of magnitude higher compared to its natural substrate α-ketoisovalerate [2] as detected for *S. marcescens*. No enzymatic data are available to our knowledge for the corresponding *E. coli *enzymes.

This possibility of the enzymes encoded by the *leuABCD *operon to use other substrates suggests a direct possibility for chain elongation from pyruvate (3C) over α-ketobutyrate (4C) and α-ketovalerate (5C) to α-ketocaproate (6C). This hypothesis is further supported by experimental data from Apostol et al. [16]. In their study the first response to hemoglobin induction is a decrease of the leucine pool and the accumulation of pyruvate, which results in the immediate increase of norvaline, followed by a later accumulation of norleucine.

In the actual study we show by metabolic shift experiments that norvaline is not accumulated in wild type *E. coli *K-12 strains during aerobic cultivation on mineral salt medium, but only after a switch from aerobic to anaerobic conditions at a high glucose concentration. Our results support the model proposing that pyruvate is directly converted to α-ketobutyrate, which indicates that also conditions which lead to accumulation of pyruvate by natural responses may cause norvaline synthesis. The results also provide evidence that norvaline synthesis after an oxygen downshift is not related to induction or derepression of the enzymes of the branched chain amino acids, but rather to a metabolic overflow reaction.

## Results

Earlier it has been suggested that norvaline synthesis may be triggered by conditions which lead to accumulation of pyruvate [16]. It is well known that pyruvate strongly increases in *E. coli *during unlimited growth on glucose if the environment is shifted from aerobic to anaerobic conditions. However, it has not been tested so far whether such a shift also stimulates norvaline synthesis.

**Figure 2:**
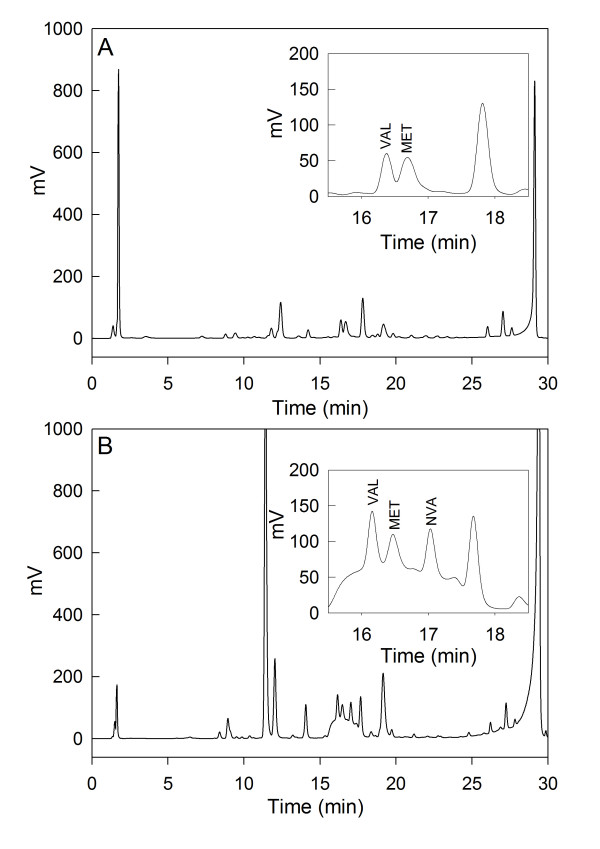
**HPLC chromatogram of crude extracts (containing cells and medium) from *E. coli *W3110 cultivations in glucose containing mineral salt medium.** Samples were harvested 10 h after an oxygen downshift (B) or during an aerobic batch culture (A) grown.

**Figure 3:**
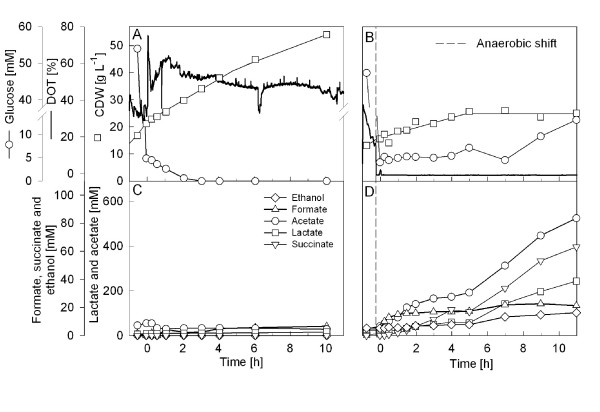
**High cell density cultures of *E. coli *W3110 without (A, C) and with a stirrer downshift resulting in oxygen limited culture (B, D).** The graphs show the data for cell dry weight (g L^-1^), dissolved oxygen tension (DOT), glucose concentration (A, B) and the anaerobic metabolites acetate, lactate, formate, ethanol, succinate (C, D, symbols shown in C). Anaerobic conditions in graphs (B, D) were caused by a downshift of the stirrer speed whereas the aeration rate was kept constant, as described in the Material and Methods section. The glucose feed was started at 0 hours. The stirrer downshift was performed at -10 min.

As the norvaline level in aerobically grown cells is below the detection limit (Fig. 2A) and difficult to analyze at low cell densities (own unpublished results), we performed cultivations at high cell densities. Therefore *E. coli *W3110 was cultivated on mineral salt medium with an initial glucose concentration of 40 g L^-1^. The culture was grown up to an OD_600 _of about 40 (about 15 g L^-1 ^cell dry weight) when oxygen limitation was initiated by a step-decrease of the agitation rate, resulting in a lower oxygen transfer rate and consequently the dissolved oxygen tension (DOT) decreased immediately to zero. The amount of glucose was kept non-limiting by constant feeding of a highly concentrated glucose solution in a fed-batch mode. As a control an aerobic cultivation was performed in the same way, however with a glucose-limited feeding to avoid oxygen limitation. As is seen from figure 3, the typical anaerobic metabolites of the mixed acid fermentation of *E. coli *accumulate after the downshift of the stirrer rate including acetate, formate, succinate, lactate, and ethanol indicating that the culture responds by anaerobic metabolism despite the continuous supply of air.

**Figure 4:**
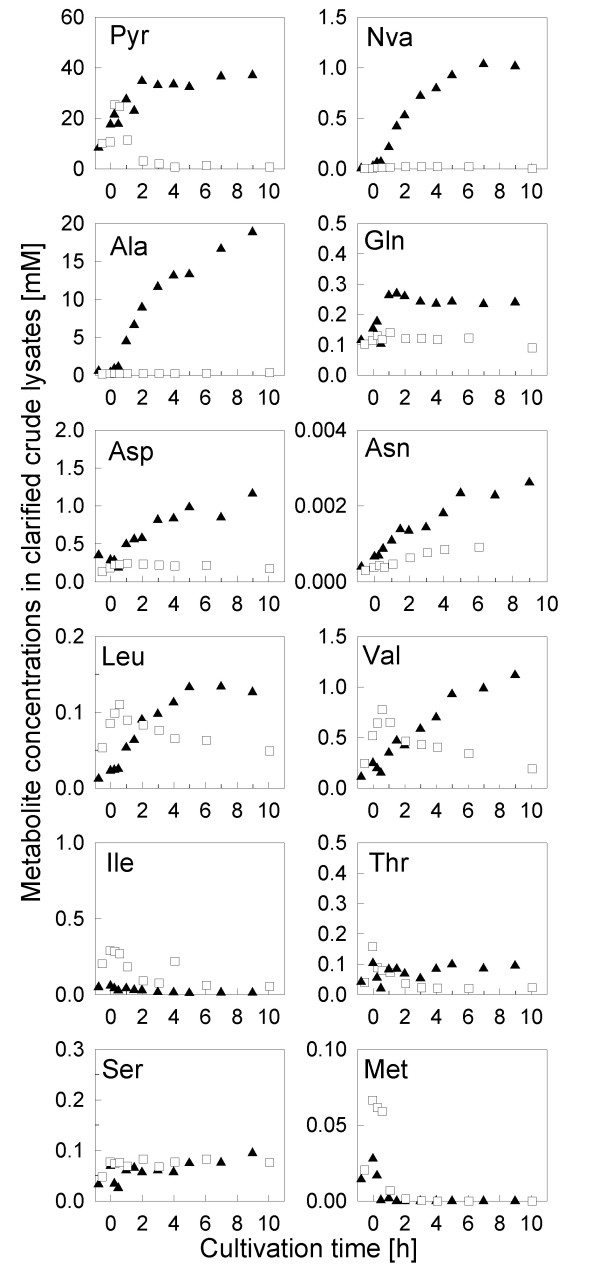
**Concentrations of free amino acids and pyruvate during fed-batch cultivations of *E. coli *W3110 without (as control, open squares) and with a stirrer downshift resulting in an oxygen limited culture (closed triangles).** The analysis was performed from clarified crude extracts containing medium and cells as described in Materials and Methods.

As expected, aside from a decrease of the cell growth, the anaerobic shift also caused accumulation of pyruvate. Pyruvate is a central metabolic compound for many pathways. Therefore one might hypothesisze that such a significant accumulation of pyruvate affects not only the flow into the typical anaerobic metabolic pathways but also into the pyruvate-connected routes for amino acid synthesis. To investigate this, we analyzed the concentrations of free amino acids in both cultivation types, with a downshift of the stirrer rate and without (Fig. 4). Interestingly, in agreement with our hypothesis we detected a significant increase in certain amino acids after the switch to anaerobic growth conditions especially in pathways which are closely connected to pyruvate, such as alanine, valine, and somewhat less significantly for leucine. Also glutamine and aspartate showed a significant increase, and asparagine increased with a similar kinetics as aspartate, but the concentration remained three orders of magnitude lower (μM range). In contrast isoleucine did not increase (see Fig. 4), and similarly most of the other amino acids, which are not directly related to pyruvate, did not increase in their concentrations (not shown).

Interestingly, a distinct norvaline peak was detected in HPLC chromatograms of the anaerobic culture which was not found in aerobic control cultures (see Fig. 2). Norvaline increased significantly and accumulated to about 1 mM at 7 hours after the stirrer downshift. This was the highest concentration of a branched chain amino acid detected during the cultivation with the stirrer downshift. In contrast, the norvaline remained below the detection limit in all samples from the aerobic control fermentation (see Fig. 4).

**Figure 5:**
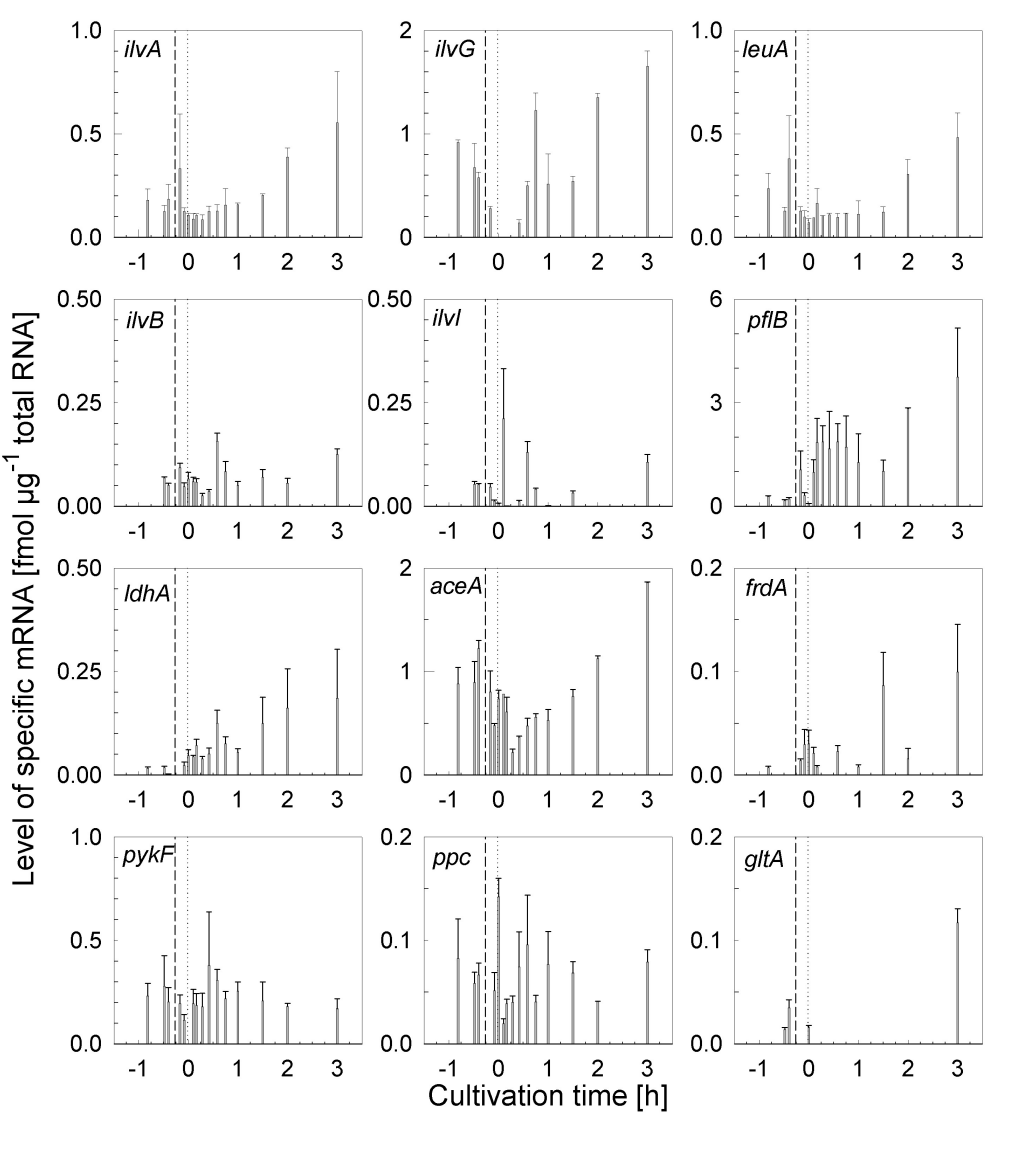
**Dynamic response of the mRNAs of a number of mRNAs after the stirrer downshift during cultivation of *E. coli *W3110.** The mRNA levels were analyzed by bead-based sandwich hybridization and quantified by use of *in vitro *standards.

**Figure 6:**
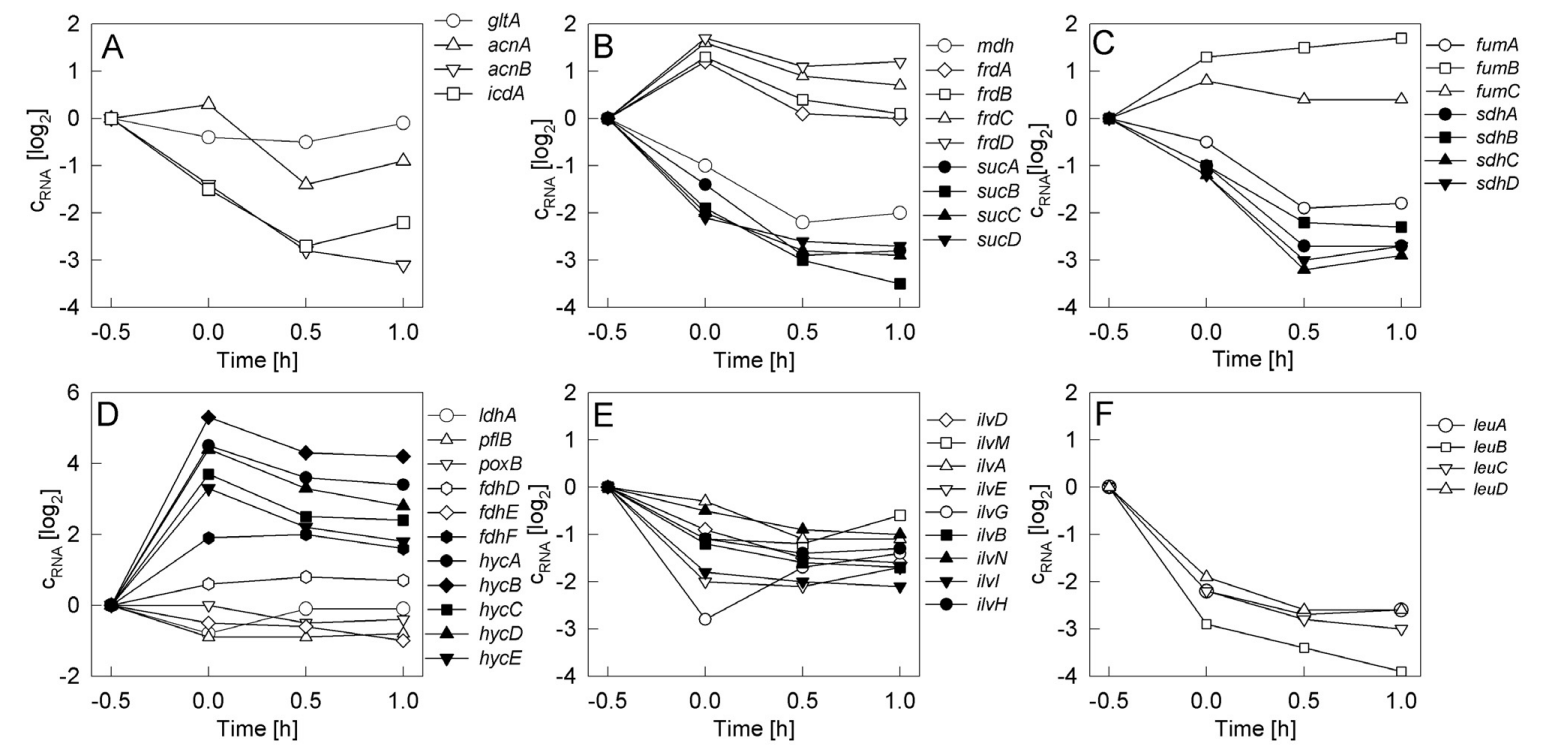
DNA microarray data on the dynamic change of the mRNAs of key enzymes of the tricarbonic acid cycle (A-C), anaerobic metabolism (D), and the branched chain amino acid pathway (E, F) during cultivation of *E. coli *W3110 with a stirrer downshift to cause anaerobic conditions.

Next, it was interesting to investigate whether norvaline accumulation occurs simply due to the accumulation of pyruvate, or whether additionally a deregulation of the synthesis of enzymes of the branched chain amino acid pathway is needed. Therefore, we studied the transcriptional profile of a number of marker genes during the shift to anaerobiosis (Figs. 5 and 6). The transcriptional profiles showed a clear induction of anaerobic metabolism by the stirrer downshift. The most significant event was the induction of pyruvate formate lyase (*pflB*), which responded immediately after the DOT dropped to zero, and interestingly even responded very sensitively to small changes in the process. For example, the *pflB *mRNA level immediately decreased when the culture run into glucose starvation for a short time at about 15 min after the anaerobic shift and it increased immediately after the DOT decreased to zero again (Fig. 5). Also the expression of lactate dehydrogenase (*ldhA*) behaved similarly. Also DNA-microarray analysis indicated a significant induction of the genes of anaerobic metabolism, such as the FNR regulon and the operons of formate dehydrogenase (*fdh*) and formate hydrogen lyase (*hyc*) which are included in formate degradation, already 20 min after the down-shift (Fig. 6). It should be remarked, however, that the *pflB *signal from the microarrays was different. In the DNA microarrays no induction of *pflB *was detected, which is remarkable as it contradicts the results with the sandwich hybridization assay and the finding that formate accumulated significantly.

A typical anaerobic reaction was the strong repression of the *aceE *and *aceF *mRNAs encoding polypeptides of the pyruvate dehydrogenase complex as observed by microarray analysis (not shown, see additional material). All other genes of the glycolysis were only marginally downregulated. Also the genes of the tricarbonic acid cycle reacted in a typical way [27,28] (see Fig. 6), the mRNAs of the *suc *and *sdh *operons decreased very strongly. A significant decrease was also observed for the *icdA *mRNA encoding for isocitrate dehydrogenase, *mdh *mRNA encoding for malate dehydrogenase, and *fumA *encoding for the aerobic fumarate hydratase. A strong increase was seen for the mRNAs of enzymes of the left branch of TCA responsible for the reactions from oxaloacetate to succinate, such as *fumB *encoding the anaerobic fumarate hydratase and the *frd *operon encoding subunits of the fumarate reductase. The mRNA of citrate synthase (*gltA*) was slightly repressed and recovered later according to the DNA array data (Fig. 6a) and similar also in the sandwich hybridization (Fig. 5), although here the *gltA *level decreased below the detection limit, but showed a high expression in the last sample.

The data for the mRNAs of the branched chain amino acid pathway from the DNA arrays showed generally a decrease of all of the mRNAs after the oxygen downshift with a fast initial response between the samples collected 20 min before the oxygen downshift and 10 min after it (Fig. 6e). A more detailed dynamic response was obtained by analysis of some representative mRNAs from the different operons by sandwich hybridization. Interestingly, but not unexpected, this showed a fast and transient accumulation of the *ilvA *mRNA after the anaerobic shift. Although the increase was only approximately two-fold, the curve showed a similar shape as for *pflB *(Fig. 5). All other mRNAs of the pathway were not observed to react so clearly and fast to oxygen depletion. However, there was a second slower continuous increase of the mRNA levels of *ilvA*, *leuA*, and *ilvG *after 1.5 hours. In contrast, there was no significant derepression observed for the level of the *ilvB *and *ilvI *mRNAs coding of the large subunits of acetohydroxy acid synthetases (AHAS) I and III respectively.

These data suggest that it is unlikely that the synthesis of norvaline is due to an extra derepression of the leucine operon, but that the existing enzymes account for the synthesis. This was further confirmed by 2-dimensional electrophoresis of the proteome which indicated no significant increase of any of the enzymes of the branched chain amino acid synthetic pathway after the anaerobic shift (data not shown).

## Discussion

The results presented here show that *E. coli W3110 *is able to produce the amino acid analogue norvaline after a shift from aerobic to anaerobic conditions in a culture on pure glucose mineral salt medium. This is new and interesting, as so far norvaline accumulation has been described only for mutant strains, e.g. for mutants of *Serratia marcescens *which are used for isoleucine production [5,7], and for recombinant *E. coli *strains which express of recombinant proteins with a high content of leucine and which consequently decrease the cellular leucine pool [16]. It is surprising that norvaline is produced by a normal environmental shift. The conditions which have been shown here to result in norvaline accumulation are relevant when shake flask cultures run into oxygen limitation. Also large- scale processes which are characterized by imperfect mixing may result in norvaline accumulation, as it is known that high glucose concentration and oxygen limitation characterize feeding zones of large-scale bioreactors [29]. Although there is no specific cellular function known for norvaline in *E. coli*, it has been shown that norvaline can be incorporated in recombinant proteins instead of leucine, which is generally not wanted.

Pyruvate is a starting metabolite for the branched chain amino acid pathway and the synthesis of the amino acid analog norvaline from α-ketobutyrate. There is strong evidence that the synthesis of norvaline or norleucine is due to direct chain elongation of pyruvate over 2-ketobutyrate and 2-ketocaproate (for norleucine) if the derepression of the leucine operon is caused by depletion of the amino acid leucine through strong induction of recombinant proteins with an over-average content of leucine [15,16]. In the case of our experimental set-up the leucine concentration was low due to cultivation on mineral salt medium and correspondingly the leucine operon was derepressed as is seen by the level of α-IPMS mRNA confirming the results by Bogosian et al. [15].

The critical factor in our experiments was the accumulation of pyruvate, caused by down-shifts of the oxygen level. Pyruvate accumulation and norvaline incorporation into a recombinant product has been observed by Apostol et al. [16] in aerobic conditions. Although the authors did not discuss the origin of this increase, it seems that the high pyruvate concentration in their cultivation resulted from changes in metabolic fluxes through the glycolysis due to expression of their recombinant product. Uncoupling of the glycolysis and respiration after strong induction of recombinant product have been observed earlier [30], but have not been attributed to fluxes in the amino acid pathways.

As the high pyruvate concentration in crude broth lysates and a derepression of the leucine operon were fulfilled in our experiments, we believe that norvaline accumulation occurred by chain elongation of pyruvate. Although a detailed metabolic flux analysis is needed for validation, this hypothesis is strongly supported by the high concentration of pyruvate, which is two orders of magnitude above the concentration of threonine and significantly above the apparent K_m _of the α-isopropylmalate synthase for pyruvate (10 mM, [24]). The direct chain elongation by the enzymes of the leuABCD operon is more likely than assuming the flux going over the long oxaloacetate and threonine pathway.

It may be remarked, that we also found an accumulation of aspartate and principally there should be the option that α-ketobutyrate is synthesized over the aspartate-threonine pathway. Interesting in this view is the anaerobic positive regulation of *ilvA*. *IlvA *mRNA was clearly shown to accumulate by measuring the dynamic response by sandwich hybridization although this response was not seen in the DNA array analysis, which only covered the first hour after the anaerobic shift with 3 samples collected in 30 min time space. However, despite the derepression of gene expression during anaerobic growth as monitored on the mRNA level (Fig. 5) and by protein synthesis (^35^S-methionine labeling of proteome and 2D electrophoresis, not shown) the amount of threonine deaminase was not significantly increasing (2D electrophoresis, not shown). Also, the level of threonine did not increase, which might be eventually expected if the flux through this pathway should be high. Therefore, we conclude that norvaline is produced by direct chain elongation of pyruvate as described before in recombinant processes.

Norvaline accumulation was only observed when we shifted the cells from aerobic to anaerobic conditions abruptly at a high glucose concentration. No norvaline accumulation was found when the aeration was shut off in a glucose limited chemostat and the glucose concentration increased gradually (data not shown), or when glucose limited cells in a chemostat growing with a specific growth rate of 0.1 h^-1 ^obtained a glucose pulse. Similar chemostat experiments performed by Chassagnole et al. [31] showed that the cellular pyruvate level only increased to about 4 mM, which is far below the level which was e.g. detected by [16] and in our studies. Long term cultivation under lower growth rate decreases the glucose uptake capacity [32] and although experimental data are not available to our knowledge, it could be proposed that the lower influx of glucose in a culture growing with a low specific growth rate may negatively affect the accumulation of pyruvate. Therefore we see the chemostat data in agreement with the assumption that a high glucose flux into the glycolysis is an important parameter.

Finally, we want to highlight the fact that the accumulation of norvaline might be favored in *E. coli *K-12 strains by the frameshift mutation in the *ilvG *gene, an interesting point, which is currently under investigation.

## Conclusion

In this study we have shown that norvaline can accumulate in the well-known *E. coli K-12 *strain W3110 if it is grown in mineral salt medium under conditions where fast growing cells undergo a shift to anaerobiosis. The important parameters seem to be a high expression of the enzymes of the leucine operon and the accumulation of pyruvate. It is likely that pyruvate is the substrate for direct keto chain elongation to α-ketobutyrate and further to α-ketovalerate from which norvaline is synthesized by transamination. The level of accumulated norvaline is very high. It remains to be investigated whether this might have relevance for the cell physiology. So far in most proteins where norvaline was incorporated the activity of the protein was maintained. The new mechanism of norvaline might be of direct biotechnological relevance if one aims to incorporate norvaline into proteins, e.g. for structural studies.

Our finding also may be of practical relevance for securing the production of non-modified recombinant proteins. The oxygen level e.g. in a shake flask culture or the occurrence of oxygen starvation zones in a large-scale bioreactor may have an impact on the appearance and incorporation of norvaline.

## Methods

### Strain and cultivation conditions

The strain used in this study was E. coli W3110 [F^-^IN(*rrnD-rrnE*)1].

The cultivations were performed in a Biostat C 15 L bioreactor with the DCU-3 controlling unit and MFCS-win supervisory system (Sartorius) with an initial working volume of 8 L. The mineral salt medium contained per liter: 14.6 g K_2_HPO_4_, 3.6 g NaH_2_PO_4 _× 2 H_2_O, 2.0 g Na_2_SO_4_, 2.47 g (NH_4_)_2_SO_4_, 0.5 g NH_4_Cl, 1.0 g (NH_4_)_2_-H-citrate, 2 mM MgSO_4_, 0.1 g thiamine hydrochloride, 0.1 mL antifoam 204 (Sigma) and 2 mL trace element solution [33]. The initial glucose concentration was 40 g L^-1^. The feed solution contained 650 g L^-1 ^glucose. 2 mL L^-1 ^of sterile filtered 1 M MgSO_4 _were added regularly per OD_600 _= 10 increase.

Two precultures were performed in LB medium and mineral salt medium with 10 g L^-1 ^of glucose without antifoam agent consecutively at 37°C at a rotary shaker at 180 rpm. Main cultivations were started as batch cultures at a temperature of 37°C. The pH was kept at 7.0 by controlled addition of 25% ammonia solution. At the end of the exponential growth phase (cell dry weight about 16 g L^-1^) the stirrer rate was lowered from 1000 rpm to 500 rpm, to provoke oxygen limitation by decreased oxygen transfer. Constant glucose feed of 100 g L^-1 ^h^-1 ^was started 15 min after the oxygen drop which was enough to ensure glucose excess during the whole cultivation.

### Analysis of cell growth

Cell growth was monitored by measurement of the absorbance (OD_600_) and cell dry weight as described earlier [34]. One unit of OD_600 _corresponds to a dry cell weight of 0.44 g L^-1^.

### Amino acid analysis

The amino acids were analyzed from clarified crude broth lysates. Therefore broth samples, containing medium and cells, were taken and immediately shock-frozen in liquid nitrogen and stored at -20°C until the sample preparation. Samples, diluted to same cell density with 0.9% NaCl solution, were sonicated on ice for 2 × 30 sec with 30 sec cooling break between the sonication steps. After sonication the cell debris was removed by centrifugation for 10 min (+4°C, 16,100 × g) and the supernatant was purified from macromolecules by centrifugation for 60 min at 14,000 × g and +4°C using Microcon 3 kDa cut-off membranes (Millipore). The filtrate was diluted 20 times with 0.1 M HCl and OPA – precolumn derivatisation was used for the amino acid analysis. OPA reagent (Agilent) was diluted 5 times in 0.4 M borate buffer (Agilent) and equal amounts of diluted sample and OPA reagent were mixed for reaction. After approximately 1 min 20 μL of derivatisation reaction solution was injected into the Zorbax Eclipse column (Agilent). The inorganic eluent contained 0.03 M sodium acetate, 0.25% tetrahydrofurane (v/v) and 100 ppm sodium azide, and the pH was set to 7.2 with 1% acetic acid [v/v]. The organic eluent consisted of 80% acetonitrile and 20% of 0.03 M sodium acetate [v/v]. The HPLC equipment consisted of pump series (P580), autosampler (ASI-100), column oven (HTS-585) and fluorescence detector (RF-2000) from Dionex.

### Analysis of metabolites and glucose

Acetate, pyruvate, formate, succinate, lactate, ethanol, and glucose were analyzed from medium samples, which were immediately centrifuged for 3 min at 16,100× g and +4°C. The supernatant was filtered (0.2 μm) and frozen in liquid nitrogen. Pyruvate was analyzed from clarified crude extracts which were prepared as described for amino acid analysis. The metabolites were analyzed in a Merck-Hitachi HPLC system (Model D-7000) with an ICSep COREGEL 87H3 column. An L-4259 UV-VIS detector (Merck-Hitachi) at 210 nm was used for the organic acids and a differential refractometer RI-71 (Merck) for glucose and ethanol.

### mRNA analysis by sandwich hybridization

Samples for mRNA analysis were collected as described earlier [34]. Samples were shortly mixed by vortexing, divided in 0.5 mL aliquots and centrifuged for 3 min at 16,100 × g and +4°C. The pellets were resuspended in 250 μL of RNALater (Ambion, USA) and stored at -20°C until analysis. RNA was extracted using the total RNA kit (A&A Biotechnology, Poland) following the instructions of the kit. A sandwich hybridisation assay was performed as described earlier [35]. *In vitro *transcribed RNA molecules were used as standards for quantification of mRNA molecules in the samples (see table 1 for the primers used). The amplification of *in vitro *RNA was done as described earlier (28). For each gene of interest a detection probe labeled with digoxigenin (Roche, USA), a capture probe labeled by biotin (by probe manufacturer), and two unlabelled helper probes were designed. Different sets of probes were tested and the set producing the highest signal in the sandwich assay was chosen (see Table 1 for details of the probes).

**Table 1:** Primer and probe sequences used in this study.

***Probe name***	**Sequence (5'-3')**	**Position/Modification**
***ilvA***		
Forward primer	TGTCGTCGCGTCTTGATAAC	119
Reverse primer	TTCGTCGTGGCAATCGTAGC	1506c
Helper probe 1	GGAAGGTTTCGTCACCGATG	757c
Detection probe	GTCGAGATACTCCTGGCATA	780c [3' DIG tail]
Capture probe	TCGCTATCGACGGTGATGAT	803c [5' biotin]
Helper probe 2	TCCTTCATCGCCGCACAGAT	827c
***ilvG***		
Forward primer	ACTTGCTATGCCGACATGAG	122
Reverse primer	GGCCAGACGTTCTCAAGTTC	1593c
Helper probe 1	CCAGAAAGCTGTGCTTGGTA	382c
Detection probe	CAACTCTTCCAGCGATGCA	402c [3' DIG tail]
Capture probe	AATGCTTCAGCCATGATGCG	425c [5' biotin]
Helper probe 2	ACGACCTGAGCAGGCAACGT	447c
***leuA***		
Forward primer	CACATTGCGCGACGGTGAAC	30
Reverse primer	CTGCGGCACGCCAGATATTG	1510c
Helper probe 1	TGCTTACCGATGAAGGCCAG	1151c
Detection probe	AATGCTCCGGCTCTTCTTGC	1171c [3' DIG tail]
Capture probe	CGCTGAAGTAATCCAGATGG	1192c [5' biotin]
Helper probe 2	ATCGTTAGAGCCAGACTGCA	1212c
***ilvB***		
Forward primer	GCGGTTCTATGCTGCCTGTT	107
Reverse primer	GCGGATCGGCTTCGTTATTC	1561c
Helper probe 1	GCGTTGATCAGGCCGTAA	1127c
Detection probe	TCATCGACACAGGCGGCAA	1148c [3' DIG tail]
Capture probe	CGTCGGTGGTGATAATTGCA	1171c [5' biotin]
Helper probe 2	TCCACATCTGATGCTGACCA	1192c
***ilvI***		
Forward primer	TTGTCTGGAGCCGAGATGGT	10
Reverse primer	CATGTCCTGCCACTGCTTCA	1461c
Helper probe 1	TTGTTCGAGGACCTGGC	990c
Detection probe	GCGGATTCTTGCGACAAG	1019c [3' DIG tail]
Capture probe	CTCATCCAGTGGTTGATG	1038c [5' biotin]
Helper probe 2	TTGCTGCCACCAGTC	1059c
***pflB***		
Forward primer	TTCCTGGCTGGCGCTACTGA	128
Reverse primer	TCCATCGCGTCGAGCAGCAT	2162c
Helper probe 1	AGGTAGCCGAAGTAAGTCCA	791c
Detection probe	TCTGAGACTTAACAGCAGCC	811c [3' DIG tail]
Capture probe	CGAAGGACATTGCAGCACCG	832c [5' biotin]
Helper probe 2	CAGGAAGGTGGAGGTACGAC	852c
***ldhA***		
Forward primer	CTGCAACAGGTGAACGAGTC	46
Reverse primer	CTGGATCACGTCGTTGGATT	831c
Helper probe 1	GGCTGGAACACGGACTACTT	297c
Detection probe	CATCATACCGATGGCGTGTT	339c [3' DIG tail]
Capture probe	GCAACGGCCTCTGGATCATA	317c [5' biotin]
Helper probe 2	AATACGGCGGTTCAGCGTCA	360c
***aceA***		
Forward primer	CATACAGTGCGGAAGATGTG	77
Reverse primer	GAACTGCGATTCTTCAGTGG	1302c
Helper probe 1	ACCACAGCTGGCACCGAGTT	362c
Detection probe	ACCATTGGATCTGATCGGCA	409c [3' DIG tail]
Capture probe	ACGGAAGGTGTTGTTGATCC	387c [5' biotin]
***frdA***		
Forward primer	GCCGATCTTGCCATTGTAGG	16
Reverse primer	TGGCGGCAGCGTAGTAATCT	1728c
Helper probe 1	TTCATTGCTACCAGGCCGCG	524c
Detection probe	CCAGCGTGCCTTCCATCATG	544c [3' DIG tail]
Capture probe	CGCGTTAGCACGGATCTGCA	564c [5' biotin]
Helper probe 2	CCGCCAGTAGCCATAACGAC	584c
***pykF***		
Forward primer	GAATCTGCGCAACGTGATGA	144
Reverse primer	TGTTAGTAGTGCCGCTCGGT	1390c
Helper probe 1	CCTTCATACGTTACCGCAAC	335c
Detection probe	GCCAACAGACAGGTCAGTAG	360c [3' DIG tail]
Capture probe	GATCAGACCATCGTCAACCA	390c [5' biotin]
Helper probe 2	CTTCAATGGCGGTAACTTCC	415c
***ppc***		
Forward primer	GCGCTGGCAATGATGCTAAC	137
Reverse primer	CTCGGCCTGCAATACGTTCA	2535c
Helper probe 1	CCTGGCAGGTATCGAGCACT	1378c
Detection probe	ATGGAGCCTTGCGGTGCTTC	1433c [3' DIG tail]
Capture probe	TTCGCCATCGAGATCACGTA	1406c [5' biotin]
Helper probe 2	TGGACAGCCAGTACGTCGGA	1460c
***gltA***		
Forward primer	CTTCACTTCAACCGCATCCT	138
Reverse primer	GGCAATCTTCATACCGTCAC	1221c
Helper probe	CCATAGCACGTTCCAGAATC	661c
Detection probe	GGTAGAGGCGTTCTGTTCAT	708c [3' DIG tail]
Capture probe	GTGCAGGATCAGAATACGGT	681c [5' Biotin]

The T7-promoter sequence CTAATACGACTCACTATAGGGAGA was added in the 5'-end of each forward primer.

### Microarray analysis

Experimental procedures for GeneChip (Affymetrix, Santa Clara, CA, USA) were performed according to the Affymetrix GeneChip Expression Analysis Technical Manual. Affymetrix *E. coli *Genome 2.0 array was used. Sample preparation was equal to mRNA analysis by sandwich hybridization described in previous chapter.

The microarray data have been deposited in NCBI's Gene Expression Omnibus [36] and are accessible through GEO Series accession number GSE12006 .

## Competing interests

The authors declare that they have no competing interests.

## Authors' contributions

JS carried out the experiments and drafted the manuscript. CF participated in the experiments with the anaerobic shift simulator and performed part of the SH mRNA analyses. CL and JB performed the 2D electrophoresis experiments. JV performed the DNA arrays. PN conceived of the study, and participated in its design, coordination, and drafting of the manuscript. All authors read and approved the final manuscript.
